# Impact of the *MDM2* splice-variants *MDM2-A*, *MDM2-B* and *MDM2-C* on cytotoxic stress response in breast cancer cells

**DOI:** 10.1186/s12860-017-0134-z

**Published:** 2017-04-17

**Authors:** Johanna Huun, Liv B. Gansmo, Bård Mannsåker, Gjertrud Titlestad Iversen, Jan Inge Øvrebø, Per E. Lønning, Stian Knappskog

**Affiliations:** 1 Section of Oncology, Department of Clinical Science, University of Bergen, 5020 Bergen, Norway; 2 Department of Oncology, Haukeland University Hospital, Bergen, Norway; 3 Department of Biology, University of Bergen, Bergen, Norway; 4 Present address: Department of Oncology and Palliative Medicine, Bodø, Norway; 5 Present address: Huntsman Cancer Institute, University of Utah Health Care, Salt Lake City, USA

**Keywords:** MDM2, *MDM2* splice variants, MDM2-A, MDM2-B, MDM2-C, Breast cancer, Doxorubicin

## Abstract

**Background:**

The murine double minute 2 (MDM2) is an oncogene and a negative regulator of the tumor suppressor protein p53. *MDM2* is known to be amplified in numerous human cancers, and upregulation of MDM2 is considered to be an alternative mechanism of p53 inactivation. The presence of many splice variants of *MDM2* has been observed in both normal tissues and malignant cells; however their impact and functional properties in response to chemotherapy treatment are not fully understood.

Here, we investigate the biological effects of three widely expressed alternatively spliced variants of *MDM2*; MDM2-A, MDM2-B and MDM2-C, both in unstressed MCF-7 breast cancer cells and in cells subjected to chemotherapy. We assessed protein stability, subcellular localization and induction of downstream genes known to be regulated by the MDM2-network, as well as impact on cellular endpoints, such as apoptosis, cell cycle arrest and senescence.

**Results:**

We found both the splice variants MDM2-B and -C, to have a much longer half-life than MDM2 full-length (FL) protein after chemotherapy treatment indicating that, under stressed conditions, the regulation of degradation of these two variants differs from that of MDM2-FL. Interestingly, we observed all three splice variants to deviate from MDM2-FL protein with respect to subcellular distribution. Furthermore, while MDM2-A and -B induced the expression of the pro-apoptotic gene *PUMA*, this effect did not manifest in an increased level of apoptosis.

**Conclusion:**

Although MDM2-B induced slight changes in the cell cycle profile, overall, we found the impact of the three *MDM2* splice variants on potential cellular endpoints upon doxorubicin treatment to be limited.

**Electronic supplementary material:**

The online version of this article (doi:10.1186/s12860-017-0134-z) contains supplementary material, which is available to authorized users.

## Background

The E3 ubiquitin ligase Murine Double Minute 2 (MDM2) is the key negative regulator of the p53 tumor suppressor protein. MDM2 binds and ubiqutinates p53, facilitating its proteasomal degradation [1–4]. p53, on the other hand, can induce transcription of *MDM2*, generating a negative feedback loop [5, 6]. Furthermore, amplification and overexpression of *MDM2* have been implicated in various types of cancer [1, 7, 8].

The *MDM2* gene consists of 12 exons encoding 491 amino acids [9]. MDM2 has a well characterized p53 binding domain at the N-terminal and a highly conserved RING domain at the C-terminus, responsible for the E3 ligase activity [10–13]. Additionally, MDM2 contains a well-defined nuclear localization signal (NLS), a nuclear export signal (NES) and a nucleolar localization signal (NoLS), responsible for MDM2 localization both in the nucleus and in the cytoplasm [14].

Two decades ago, the first alternatively spliced MDM2 transcript was identified in human tumors. To date 72 different *MDM2* splice variants have been identified in human cancer and normal tissue [9, 15–18]. The presence of *MDM2* splice variants has been observed in both normal tissues and malignant cells, yet their functional properties are not fully understood. Several studies have attempted to determine whether the splice variants contribute to tumor formation or if they are expressed as a consequence of cancer progression. However, the finding that expression of *MDM2* splice variants increase upon genotoxic stress suggests that they might have a potential role in the response to chemotherapy treatment [19].

**Fig. 1:**
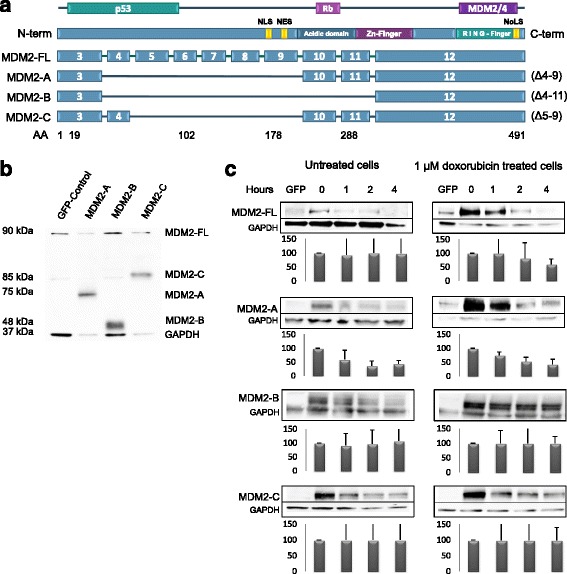
(**a**) Schematic representation of MDM2 and the splice variants MDM2-A, -B and -C. MDM2 consists of 491 amino acids. Localization of the p53, pRb and MDM2/4 binding sites, NLS, NES, NoLS, the acidic domain, the Zn-finger domain and the RING-finger domain are indicated, as well as the exon distribution. (**b**) Validation of protein expression. MCF-7 breast cancer cells transfected with pCMV-GFP control (GFP-control), MDM2-A (75 kDa), -B (48 kDa) and -C (85 kDa) analyzed 24 h post transfection. GAPDH was used as loading control. Primary antibodies were Anti-MDM2 (N-20) Sc-813 (Santa Cruz) and GAPDH (SantaCruz). (**c**) Protein stability of the expressed splice variants. MCF-7 cells transfected with MDM2-FL, MDM2-A, -B and -C at 0, 1, 2 and 4 h post cycloheximide treatment, respectively. In addition to cycloheximide treatment, *left panel* shows cells without doxorubicin treatment, right panel shows cells treated with 1 μM doxorubicin for 24 h. Primary antibodies Anti-MDM2 (N-20) Sc-813 (Santa Cruz) and GAPDH (SantaCruz). Histograms under immunoblots represent averages of triplicate experiments and show levels of the MDM2-variants relative to GAPDH-levels for each sample

So far, MDM2-A (ALT2), MDM2-B (ALT1) and MDM2-C (ALT3) are the three most commonly detected and extensively studied splice variants of *MDM2*. Each variant is observed in several types of cancer, including breast cancer [18, 20–22]. MDM2-A lacks exon 4–9, while -B and -C are lacking exons 4–11 and exons 5–9, respectively (Fig. 1a). Thus, MDM2-A, -B and -C all have the p53 binding site at the N-terminal spliced out, but the RING-finger binding domain is still present at the C-terminal, making them capable of binding to MDM2-FL.

MDM2-A has been characterized as an activator of p53, inhibiting growth in a p53-dependent manner, and to cause a decrease in the transformation and tumorigenesis in vitro [23]. Contrasting this, the same variant has also been shown to induce increased expression levels of Cyclin D1 and E, hence, suggesting that this splice variant has a tumor promoting activity in vivo [24].

MDM2-B is the splice variant most commonly overexpressed in human tumors [9]. MDM2-B is known to interact with MDM2-FL and sequester it in the cytoplasm, leading to inhibition of the MDM2-FL-p53 interaction and thereby causing stabilization and transactivation of p53 and induction of cellular growth arrest [22, 25–27]. In addition MDM2-B seems capable of inducing p53-independent cell growth [28]. Expression of MDM2-B is also shown to have tumor promoting activity by causing increased levels of Cyclin D1 and E in vivo [24].

MDM2-C is by far the least studied splice variant of the three, however -C is also known to bind MDM2-FL and has been shown to have an effect on cellular transformation independent of p53 [29].

In the present study, we aimed to investigate the potential roles of the three MDM2 splice variants MDM2-A, -B and -C in breast cancer cells in response to cytotoxic stress induced by chemotherapy. Thus, we conducted comprehensive molecular and cellular analyses in order to identify functions similar to, or differing from the well-established functions of the MDM2-FL protein.

## Methods

### Expression vectors

The sequences encoding MDM2-FL and the respective splice variants; MDM2-A, -B and -C were assembled from synthetic oligonucleotides and cloned into E.coli expression vectors (Geneart Life Technologies). *MDM2* encoding fragments were cut out using the BamHI and XhoI restriction sites. Following agarose gel purification the fragments were ligated into a pCMV eukaryotic expression vector (CMV-MCS-V5-6xHis-BGHpolyA in pCMV-cyto-EGFP-myc) using T4 DNA ligase. The utilized vector contained a sequence encoding an enhanced green fluorescent protein (eGFP) expressed from an independent CMV promoter region. Performing immunofluorescence, apoptosis and senescence analysis, a pcDNA3.1 V5-vector (TOPO) was used, providing a C-terminal V5-tag (Invitrogen). The plasmids were amplified in One Shot TOP10 Chemically Competent E.coli cells (Invitrogen) by Ampicillin selection, followed by colony PCR and purified using the QIAprep Spin Miniprep Kit (Qiagen). The constructed plasmids encoding MDM2-FL and splice variants were confirmed by sequencing using the BigDye1.1 system and Sanger sequencing prior large scale purification from E.coli by the HiSpeed plasmid maxi kit (Qiagen), according to the manufacturer’s instructions. The resulting stock solutions of the plasmids were validated by sequencing prior to introduction to a eukaryotic cell system.

### Cell culture, transfection and treatment

Cells were fingerprinted with AmpFISTR Profiler and Cofiler plus (Applied Biosystems by Life technologies) before use. MCF-7 (HTB-22; ATCC) breast cancer cells were cultivated in EMEM (Eagle’s minimum essential medium; ATCC), HCT116 *TP53*+/+ (CCL-247; ATCC) and HCT116 *TP53*−/− colon cancer cells were cultivated in McCoy’s medium (ATCC). The media were supplemented with 10% FBS, 2% L-Glutamine and 2% Penicillin Streptavidin (Lonza). HCT116 *TP53*−/− were a generous gift from Dr. F. Bunz, B. Vogelstein & K. W. Kinzler at John Hopkins University and Howard Hughes Medical Institute, MD. Prioritizing high transfection efficacy, transfection was performed using 1.85 μg/ml plasmid and 1.7 μl/ml Lipofectamin-2000 (Invitrogen). Cells were treated with dimethyl sulfoxide (DMSO) as negative control (same final concentration as for the parallel cells treated with 1 μM doxorubicin in DMSO).

### Sorting cells by flow cytometry and GFP- transfection efficiency

The cells were sorted and harvested by Flow Cytometry (FACS Aria, BD Biosciences) based on GFP-expression 12 h post transfection, before they were seeded and added new growth medium for further analysis. The average transfection efficiency was found to be 33, 38 and 37% for the MDM2-A, -B and -C expressing vectors, respectively (data not shown).

### Western blot analysis

Cells were harvested with Trypsin EDTA (Lonza), lysed with IPH buffer with protease- inhibitor, debris removed and the proteins were denatured by boiling in SDS buffer and loaded on 12% SDS-polyacrylamide gels (Bio Rad) with PS11 protein ladder (GeneOn). Separated proteins were transferred onto 0.2 μM nitrocellulose membranes by turbo blotting for 7 min, 2.5A and 25 V using the Bio Rad system. Unspecific protein binding were blocked by incubation in 5% non-fat milk in TBS-Tween_0.05%_ for one hour at room temperature or over-night at 4 °C. Membranes were subsequently incubated in MDM2 specific antibody Sc-813 (SantaCruz) recognizing exon 3. Anti-Actin (Thermo Scientific) or Glyceraldehyde 3-phosphate dehydrogenase (GAPDH; SantaCruz) was used as loading control. Further, washing of membranes in TBS-Tween_0.05%_, proteins bound by the primary antibody were detected by HRP-conjugated secondary antibody (Sigma). Signals were detected using SuperSignal West Femto Chemiluminescent Substrate (Thermo Scientific) and the LAS 4000 imager (GE Healthcare).

### Protein stability analysis

Twenty four hours post transfection the cells were treated with DMSO or 1 μM doxorubicin for 24 h. The cells were then exposed to 100 μg/ml cycloheximide (Sigma) and harvested after 1, 2 and 4 h. Cells were washed (ice-cold PBS), de-attached (Trypsin-EDTA) and neutralized (EMEM) followed by centrifugation and subsequent washing of the generated cell pellet in cold PBS. Proteins were released by cell lysis for 10 min in IPH buffer with protease and phosphatase inhibitors. Cell debris was removed by centrifugation at 13 000 rpm for 1 min. Protein concentration was measured by absorption measurements at 280 nm using the NanoDrop2000 (Thermo Scientific). By varying the amount of cell lysate, equal amount of total protein was denatured in Sodium dodecyl (SDS) sample buffer and boiled at 95 °C for 5 min, followed by Western blotting.

### Subcellular localization by indirect immunofluorescence

Cells were grown on glass coverslips and transfected. Forty eight hours after transfection the cells were fixed for 15 min in 3.7% formaldehyde. Cells were then permeabilized with 0.1% Triton X-100 for 10 min and blocked with 1% BSA in PBS for 30 min. The coverslips were then incubated with MDM2 specific antibody Sc-813 (Santa Cruz) for 1 h followed by AlexaFluor 647 conjugated secondary antibody (Life Technologies) for 30 min. The cells were incubated 10 min in 0.1% Hoechst 33 342 (Molecular Probes, Life Technologies) in PBS, washed in 1 × PBS and mounted with Fluka Eucitt quick hardening mounting solution (Life Technologies). Localization of the different splice variants were determined independently by three investigators, blinded to each other’s results, who analyzed >50 cells transfected with each of the *MDM2* splice variants.

### Quantitative PCR

Transfected and sorted cells were treated with 1 μM doxorubicin or DMSO (control) for 12 h. The total RNA was isolated using Trizol reagent (Life Technologies, Gaithersburg, MD) according to the manufacturer’s instructions. Single stranded cDNA synthesis was performed using 500 ng total RNA, oligo-dT — and random hexamer primers (Sigma) with Transcriptor Reverse Transcriptase (Roche) in accordance with manufacturer’s instructions. mRNA levels of MDM2 EXON3 (total MDM2), MDM2 3’UTR (endogenous MDM2) and RPLP2 (reference) were determined individually; EXON3 was amplified with the primers 5´-AACATGTCTGTACCTACTGATGGTGC-3´ and 5´-CAGGGTCTCTTGTTCCGAAGC-3´ and the hydrolysis probe 6FAM-AACCACCTCAC AGATTCC-BBQ. 3’UTR was amplified with the primers 5´-TGCTCCATCACCCATGCTAGA-3´ and 5´-TGGTGGTACATGCCTGTAATC-3´ and the hydrolysis probe 6FAM-TAGCTTGAACCCAGAAGGCGGA-BBQ, while *MDM4, RB1, E2F1, TP53, MTOR, CDK1A, ATM, PTEN, BCL2, APAF, GLB1* and *PUMA* by quantitative amplification reactions using custom made Realtime Ready plates (Roche; Configurator no: 100054567) using the LightCycler 480 instrument (Roche). LC480 Probes Master (Roche) was used as reaction mix. Reaction concentrations were 0.5 μM of each primer and 0.125 μM of each hydrolysis probe.

### Apoptosis assay: AnnexinV detection

Cells were transfected for 24 h, followed by treatment with DMSO or 1 μM doxorubicin for 24 h before they were trypsinated and washed in 1xPBS. Further, the cells were incubated 15 min at 37 °C in AnnexinV (Biotium) and Hoechst (Chemometec). The cells were washed once in AnnexinV buffer (Biotium) before they were re-suspended in AnnexinV buffer with 4% Propidium Iodid (PI; Chemometec) and analysed with the NucleoCounter 3000 (Chemometec). The analysis was repeated in three independent experiments.

### Cell cycle analysis

Transfected and treated cells were incubated 5 min at 37 °C in lysis buffer with Hoechst (Chemometec). Thereafter, cells received stabilizing buffer, before they were analyzed on a NucleoCounter 3000 (Chemometec) for DNA quantitation in three independent experiments.

### Cell proliferation assay by cell count

Exactly 30 000 transfected cells were seeded and counted with NucleoCounter 3000 after 24, 48 and 72 h respectively.

### Senescence assay: β-galactosidase staining

Cells were transfected for 24 h, followed by treatment with DMSO or 0.25 μM doxorubicin. After 7 days the cells were stained for β-galactosidase activity with the Senescence β-galactosidase Staining Kit (Cell Signaling) according to the manufacturer’s instructions. The staining of the cells was performed by incubation in staining solution for 14 h in a cultivation incubator with humidified atmosphere, without CO_2_ at 37 °C. Cells detected as blue were β-galactosidase positive cells versus un-colored, negative cells upon microscopic inspection (Nikon eclipse TS100). For all samples, presence of transfected plasmid was confirmed at the time of analysis by detection of GFP (manual inspection of cells under microscope).

### Statistics

Differences in subcellular localization were tested by Chi-square. Apoptosis, phase distribution in the cell cycle and senescence were tested by univariate analysis. All *p*-values are reported as two-sided.

## Results

In the present study, we examined the *MDM2* splice variants MDM2-A, -B and -C for their biological functions in breast cancer cells with and without chemotherapy treatment.

After generation of expression vectors for each of the three splice variants, we transfected MCF-7 cells and verified exogenous protein expression from the vectors by Western blot analyses: we detected MDM2-A, -B and -C at 75 kDa, 48 kDa and 85 kDa, respectively (Fig. 1b).

### Splice variant protein stability

The stability of the three *MDM2* splice variants expressed as proteins was determined subsequent to incubation with cycloheximide.

In untreated cells, while MDM2-A was degraded at a rate resembling MDM2-FL, and MDM2-B was degraded more rapidly than MDM2-FL, MDM2-C was degraded at a slightly slower rate. However, after doxorubicin treatment, MDM2-B in particular, but also -C was found stabilized as compared to MDM2-FL and MDM2-A (both MDM2-B and -C were stable after 4 h; Fig. 1c). Thus, our data strongly indicate that MDM2-B, and potentially also -C, are stabilized by different mechanisms than the MDM2-FL protein, upon cytotoxic stress.

### Subcellular localization

**Fig. 2:**
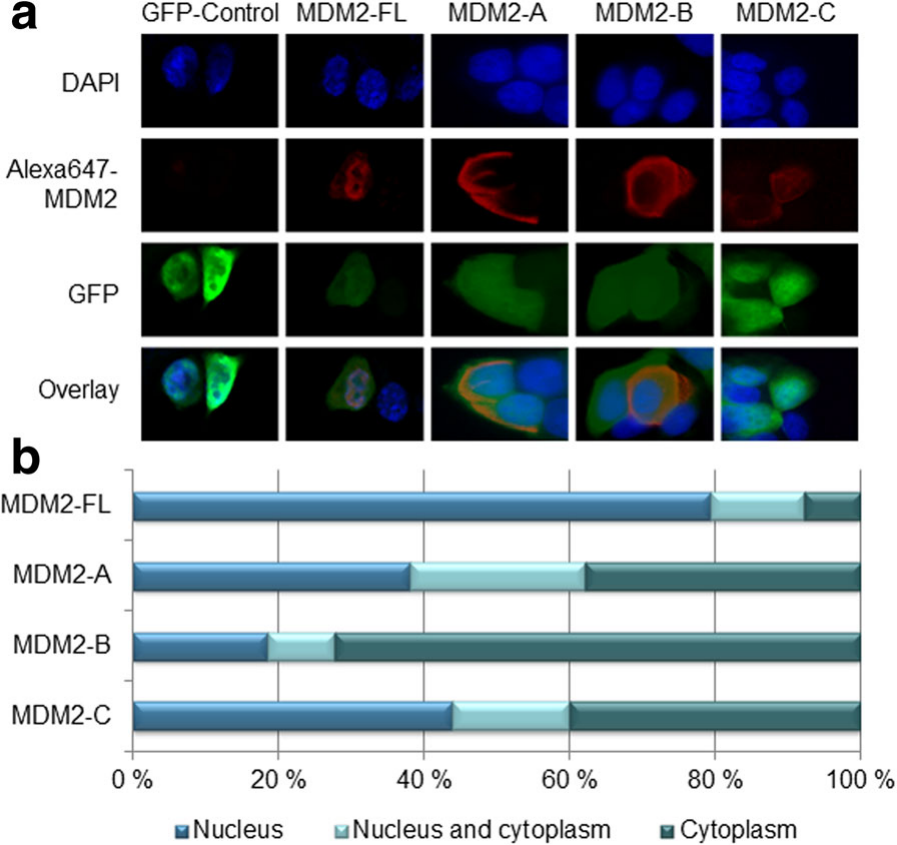
Sub-cellular localization of the MDM2-splice variants. (**a**) MCF-7 cells transfected with pCMV-GFP (GFP-control), MDM2-FL, -A, -B and -C evaluated by indirect immunofluorescence for determination of cellular localization of the proteins. *Top lane* shows the nucleus by Hoechst staining (*blue*), second lane shows the cells expressing GFP (*green*), *third lane* shows the AlexaFlour 647 MDM2 antibody (*Life Technologies, red*), and lastly, an overlay of the tree previous pictures. (**b**) Percentage of cells with exclusively nuclear (*dark blue bars*), nuclear and cytoplasmic (*blue bars*) or exclusively cytoplasmic (*light blue bars*) localization of the MDM2-A, -B and -C. For each of the differently transfected cell samples the sub cellular localization was determined for 50 transfected cells with each of the constructs. The experiment was repeated in triplicate, with three independent transfections. Cells were counted by three independent investigators blinded to sample identity and each other’s results

MDM2-FL is observed exclusively in the nucleus in the majority of cells (85%). In contrast, a different distribution pattern was observed for MDM2-A and -C; here, we observed 38 and 44% of the cells to harbor MDM2-A and -C in the nucleus only; 38 and 40% revealed cytoplasmic distribution only, while 24 and 16% respectively expressed MDM2-A and -C in both compartments (*p* < 0.001 for both MDM2-A and -C comparing distribution to MDM2-FL). For MDM2-B, the subcellular distribution was even more different from MDM2-FL, with 19% of the cells revealing a nuclear localization only, 9% revealing localization in both compartments, while as many as 72% of the cells revealed exclusively cytoplasmic distribution of MDM2-B (*p* < 0.001, compared to the MDM2-FL; Fig. 2).

### Feedback on expression of endogenous *MDM2*

We quantitated the levels of endogenous MDM2-FL mRNA upon overexpression of each of the *MDM2* splice variants.

**Fig. 3:**
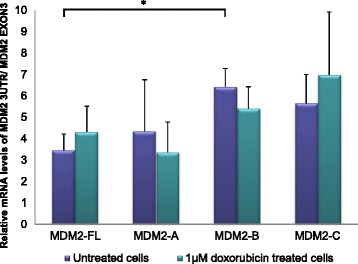
Expression of endogenous *MDM2* after transfection with the MDM2-splice variants. Relative mRNA levels of *MDM2* 3UTR / *MDM2* EXON3 (i.e. corrected for transfection efficacy) measured after MCF-7 cells were transfected with MDM2-A, -B and -C and after cell sorting by GFP expression, untreated cells (*purple bars*) or 1 μM doxorubicin treated (*green bars*) for 24 h. The experiment was repeated in triplicate, with three independent transfections. * = *p* ≤ 0.05

Endogenous levels of MDM2 mRNA remained unchanged after upregulation of MDM2-A, both before and after treatment with doxorubicin (Fig. 3). While overexpression of MDM2-B lead to a notable 1.8 fold increase in the level of endogenous MDM2-FL (*p* = 0.011), overexpression of MDM2-C caused a similar but non-significant upregulation in untreated cells. Both splice variants in addition caused a non-significant upregulation of MDM2-FL in doxorubicin treated cells.

### Regulation of potential target genes

Further, we assessed the potential influence of the splice variants on expression levels of several genes known to be regulated by MDM2 / the MDM2 network: *MDM4, RB1, E2F1, TP53, MTOR, CDK1A, ATM, PTEN, BCL2, APAF, GLB1* and *PUMA*.

**Fig. 4:**
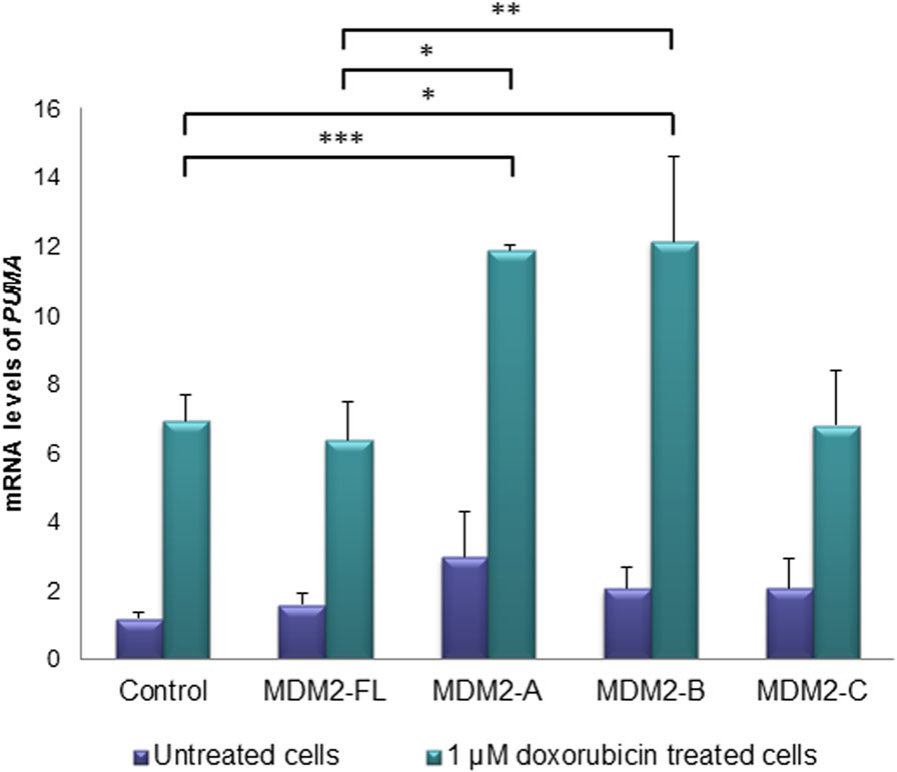
Expression of *PUMA* upon overexpression of the *MDM2* splice variants. Activation of *PUMA* after transfection with the splice variants by qPCR assay revealing relative mRNA levels of *PUMA*. MCF-7 cells transfected with pCMV-GFP (GFP-control), MDM2-A, -B and -C, sorted after 12 h based on GFP expression. Graphs show the untreated cells (*purple bars*) and the 24 h 1 μM doxorubicin (*green bars*). The experiment was repeated in triplicate, with three independent transfections. * = *p* ≤ 0.05, ** = *p* ≤ 0.01, *** = *p* ≤ 0.001

While overexpression of the three splice variants had no effect or minor a influence on most of the examined genes only, we found overexpression of MDM2-A and -B under doxorubicin treatment both to lead to an approximately two-fold increase in the induction of the pro-apoptotic gene *PUMA*, as compared to the control cells (*p* = 4.0×10^−4^ and *p* = 0.024, respectively; Fig. 4). A similar effect was not observed for MDM2-FL or -C.

### Induction of apoptosis

**Fig. 5:**
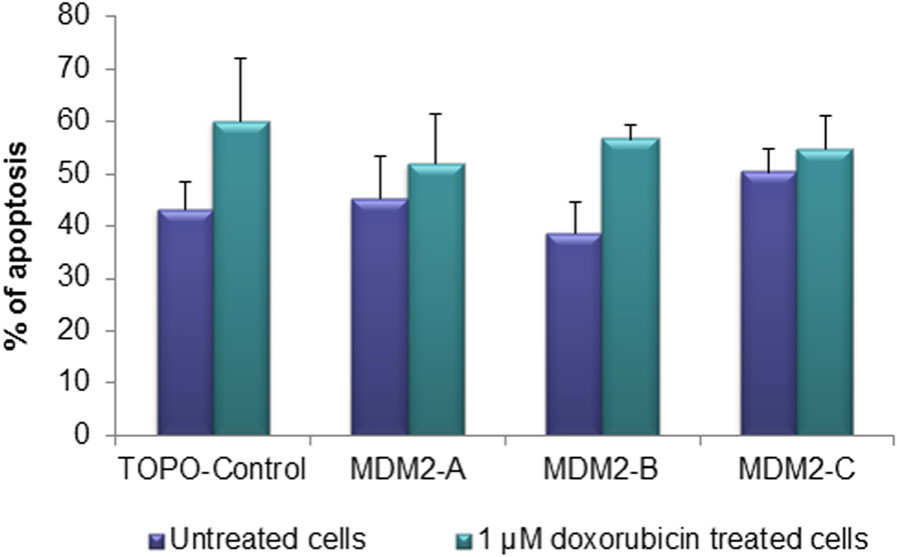
Induction of apoptosis. Graphs show the percentage of apoptotic cells after transfection with pCMV (TOPO-Control) and the splice variants. Untreated cells (*purple bars*) and cells treated with 1 μM doxorubicin (*green bars*) were analyzed by AnnexinV assay 48 h post transfection. Each pillar represents the total of apoptotic and early apoptotic cells. The experiment was repeated in triplicate with three independent transfections

None of the three splice variants had any influence on apoptosis assessed by AnnexinV, neither in untreated cells nor subsequent to doxorubicin exposure (Fig. 5). Since this was somewhat surprising, given the observed upregulation of *PUMA* in MDM2-A and -B overexpressing cells, we sought to validate this finding in additional cell lines. We therefore repeated these experiments in isogenic versions of HCT116 colon cancer cells, differing with respect to their *TP53* status only (one version being *TP53*+/+ and the other one being *TP53*−/−). Neither overexpression of MDM2-A, -B or -C had any significant effect on apoptosis either in untreated cells or after exposure to doxorubicin in either cell line (Additional file 1).

### Impact on cell cycle progression

**Fig. 6:**
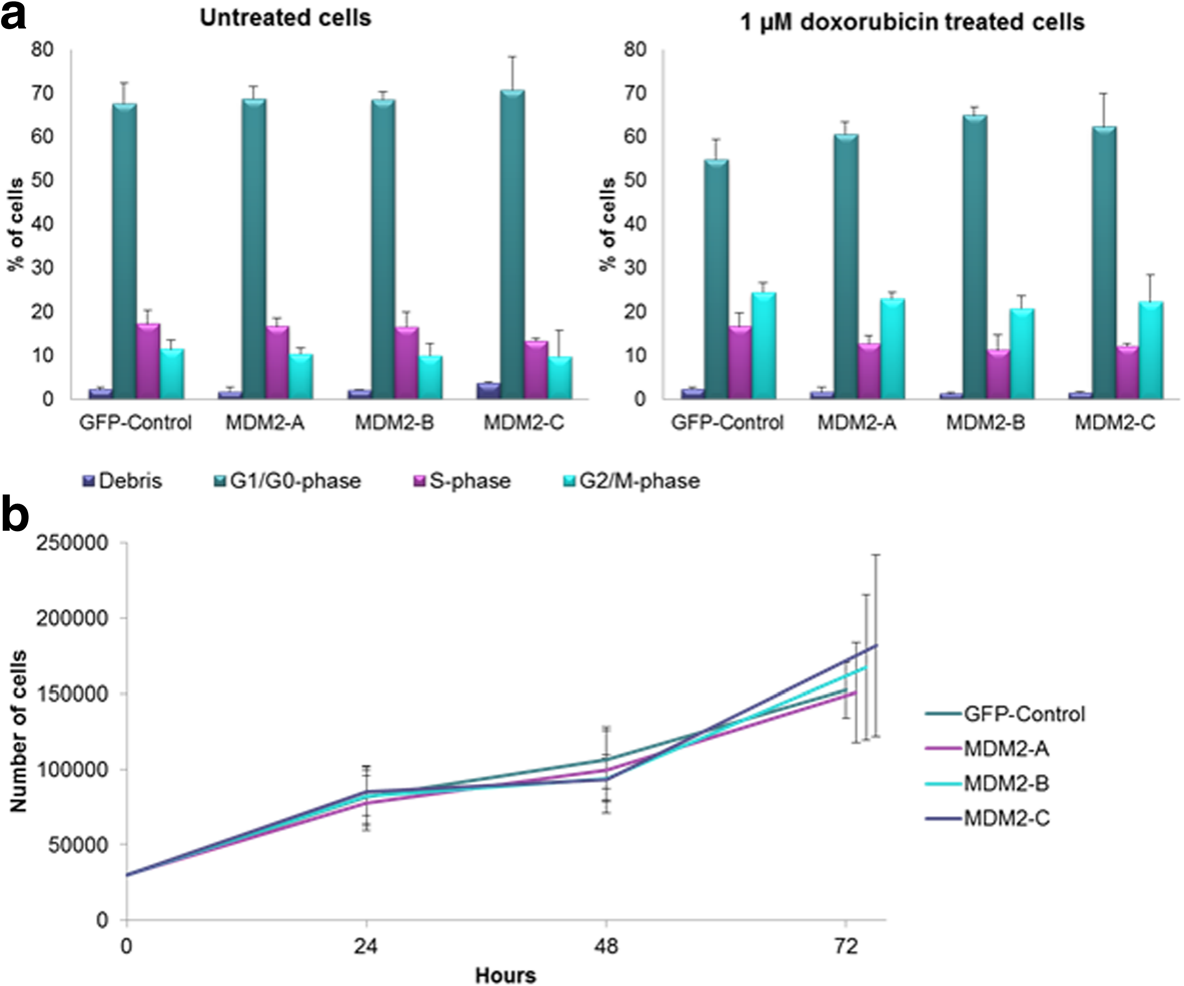
Cell cycle analysis. (**a**) MCF-7 breast cancer cells were transfected with pCMV-GFP (GFP-control), MDM2-A, -B and -C. DMSO control cells (*left graph*) or cells treated with 1 μM doxorubicin (*right graph*) for 24 h. The cells were analyzed by NucleoCounter-3000 for status of cell cycle progression. Bars represent cell debris (*purple bars*), cells in G1/G0-phase (*green bars*), cells in S-phase (*pink bars*) and cells in G2/M-phase (*blue bars*) respectively. (**b**) Cell proliferation analyzed by cell count. MCF-7 breast cancer cells transfected with pCMV-GFP (GFP-control), MDM2-A, -B and -C were counted to examine the cell proliferation 24, 48 and 72 h after transfection (data points at 72 h have been slightly shifted on the x-axis of the graph, for clarity). *Both* the experiments were repeated in triplicate, with three independent transfections

Addressing the effect of the *MDM2* splice variants on cell growth, we found no effect of overexpression of either MDM2-A, -B, or -C in untreated cells (Fig. 6a).

In contrast, doxorubicin treatment of cells overexpressing MDM2-B increased the fraction of cells in G1/G-phase (*p* = 0.044) with a corresponding decrease in S-phase (*p* = 0.022) as compared to the doxorubicin treated control cells (Fig. 6b), indicating a potential role for this splice variant in cell cycle regulation of stressed cells. While similar effects were observed for both MDM2-A and -C, the differences did not reach statistical significance.

### Activation of senescence

**Fig. 7:**
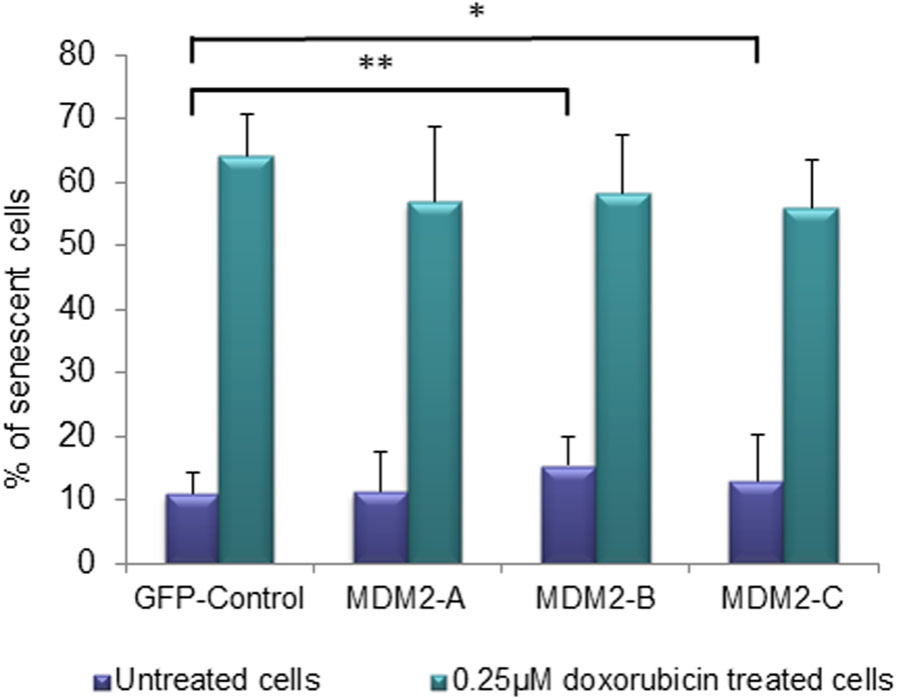
Induction of senescence. Graphs show the percentage of senescent cells after transfection with pCMV (TOPO-Control) and the splice variants, untreated (*purple bars*) or treated with 0.25 μM doxorubicin (*green bars*). Cells were analyzed by β-galactosidase assay 8 days post transfection. The experiment was repeated in triplicate with three independent transfections

In cells not exposed to doxorubicin, overexpression of MDM2-B and -C both lead to an increase in the number of senescent cells (*p* = 0.004 and *p* = 0.012, respectively), while overexpression of MDM2-A lead to a smaller, non-significant increase (Fig. 7). No such effect was observed in doxorubicin treated cells.

## Discussion

p53 is activated in response to genotoxic stress and contributes to various processes including apoptosis, cell cycle arrest and senescence [30]. MDM2 is the major regulator of p53, and overexpression of *MDM2* is considered to be a mechanism of direct p53 inactivation [31, 32]. While several splice variants of *MDM2* are identified in tumor- as well as normal cells [33], their functional effects are poorly understood. In the present study we investigated the functional effects of overexpressing the alternative spliced variants MDM2-A, -B and -C in response to chemotherapy treatment.

In line with previous findings [26, 29, 34], we found all three splice variants to be stably expressed at the protein level. Here, we show that upon cytotoxic stress, MDM2-B, and potentially also -C, might be stabilized by different mechanisms than the MDM2-FL protein, suggesting that a potential functional role of these splices might be related to cellular stress response.

Interestingly, MDM2-B was found to have a most distinct subcellular localization, differing not only from MDM2-FL, but also from MDM2-A and -C. We found a large majority of MDM2-B exclusively located in the cytoplasm, possibly indicating a distinct function in the cell. This corresponds to previous studies, finding MDM2-B to be localized in the cytoplasm in H1299 cells (lung carcinoma) and NIH/3 T3 cells (mouse fibroblasts) [24]. There are, however, contradictory findings in mouse embryonic fibroblasts, showing exogenously expressed MDM2-B predominantly to be localize in the nucleus [35]. Regarding MDM2-C, this variant is previously found in both the nucleus and in the cytoplasm in MANCA (Burkitt lymphoma), T47D (breast cancer) and also in MCF-7 cells [29], in line with our observations in the present study. Notably, MDM2-A, -B and -C are all known to bind and co-localize with MDM2-FL in the nucleus [29, 35], but MDM2-B is also found to bind to MDM2-FL in the cytoplasm making MDM2-FL unable to enter the nucleus and inhibit p53 in unstressed MCF-7 cells [26].

Strikingly, we found significantly increased levels of endogenous MDM2-FL mRNA as a result of MDM2-B overexpression. This was not the case for MDM2-A and -C overexpression. Although the mechanism behind this upregulation remains unknown, we may speculate that, since we find MDM2-B mostly located in the cytoplasm, it could bind to the MDM2-FL there, making the MDM2-FL-protein unable to enter the nucleus and inhibit p53. This could in turn affect the feedback-loop between p53 and MDM2, and cause MDM2-FL levels to increase as p53 is accumulated in the nucleus, as previously shown by others [26].

Interestingly, *PUMA*, one of the major genes leading to activation of apoptosis, was significantly upregulated at the mRNA level, after MDM2-A and -B overexpression and doxorubicin treatment. Given that PUMA is a direct transcriptional target for p53, it is likely that the MDM2-splice variants’ impact on PUMA levels is mediated via p53. Recent studies have indicated that MDM2-B has a functional role of in tumor cells as response to cellular stress (UV and Cisplatin) through activation of *p21* rather than *PUMA* [36], however, we found no change in *p21* level after overexpression of MDM2-B.

Based on our findings of *PUMA* upregulation that could indicate a role for MDM2-A and MDM2-B in apoptosis, together with previous findings indicating that MDM2-A may by associated with aging and senescence [34, 37], we assessed the impact of the splice variants on potential cellular endpoints upon doxorubicin treatment: apoptosis, growth/cell cycle progression and senescence. However, none of the three *MDM2* splice variants seemed to have any impact on activation of apoptosis or senescence, neither in untreated cells nor doxorubicin treated cells. Although this may seem contradictory to the finding of upregulated *PUMA*, notably, p53’s functions as a transcription factor and as an inducer of apoptosis have, in several systems been shown to be independent [38, 39].

Regarding cell cycle distribution, we found that the cell cycle profile changed in the chemotherapy treated cells with up-regulated MDM2-B. This could potentially be explained by MDM2-B’s ability to bind MDM2-FL in the cytoplasm (as discussed above), making MDM2-FL unable to enter the nucleus for inhibition of p53. In turn this could lead to accumulation of p53 that may trigger arrest of cells in the G1/G0-phase. Regarding MDM2-A, a previous studie has found this splice variant to inhibit growth in a p53-dependent manner [23], however, in the present study, we observed no change in cell cycle distribution as a result of overexpressing MDM2-A.

## Conclusions

We found the impact of the three *MDM2* splice variants on potential cellular endpoints upon chemotherapy treatment of breast cancer cell lines to be limited.

## Additional file

Additional file 1: Induction of apoptosis in HCT116 *TP53*+/+ and *TP53*−/−. Graphs show the percentage of apoptotic cells after transfection with pCMV (TOPO-Control), MDM2-FL and the splice variants, untreated (purple bars) or treated with 1 μM doxorubicin (green bars) analyzed by AnnexinV assay 24 h post transfection. Each pillar represents the total of apoptotic and early apoptotic cells. The experiment was repeated in triplicate with three independent transfections. (TIF 99 kb)

## Abbreviations

ATCC: American Type Culture Collection
BSA: Bovine serum albumin
DMSO: Dimethyl sulfoxide
EMEM: Eagle’s minimum essential medium
FBS: Fetal bovine serum
GAPDH: Glyceraldehyde 3-phosphate dehydrogenase
GFP: Green fluorescent protein
HRP: Horse radish peroxidase
MDM2: Murine Double Minute 2
MDM4: Murine double minute 4
PI: Propidium Iodid
SDS: Sodium dodecyl
WT: Wild-type
