# Supplementary figures and images for: Impact of the MDM2 splice-variants MDM2-A, MDM2-B and MDM2-C on cytotoxic stress response in breast cancer cells
Source: BMC Cell Biol. 2017 Apr 17;18:17. doi: 10.1186/s12860-017-0134-z (PMC5393014; doi:10.1186/s12860-017-0134-z)

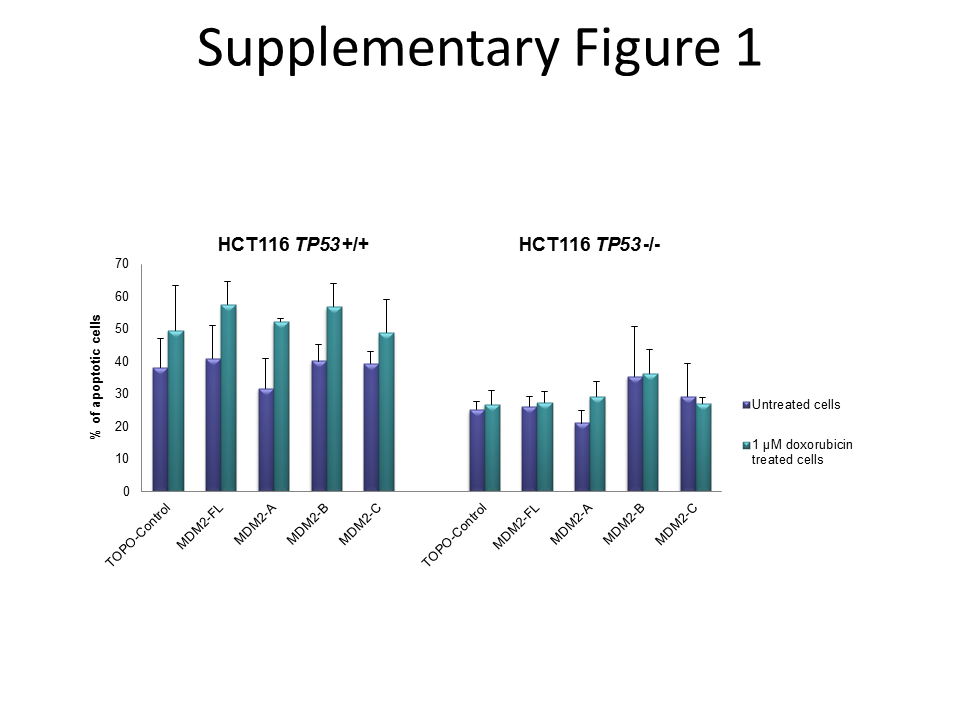

Supplement: Additional file 1: — Induction of apoptosis in HCT116 TP53+/+ and TP53−/−. Graphs show the percentage of apoptotic cells after transfection with pCMV (TOPO-Control), MDM2-FL and the splice variants, untreated (purple bars) or treated with 1 μM doxorubicin (green bars) analyzed by AnnexinV assay 24 h post transfection. Each pillar represents the total of apoptotic and early apoptotic cells. The experiment was repeated in triplicate with three independent transfections. (TIF 99 kb) [file 12860_2017_134_MOESM1_ESM.tif]
